# Realization of a complete Stern-Gerlach interferometer: Toward a test of quantum gravity

**DOI:** 10.1126/sciadv.abg2879

**Published:** 2021-05-28

**Authors:** Yair Margalit, Or Dobkowski, Zhifan Zhou, Omer Amit, Yonathan Japha, Samuel Moukouri, Daniel Rohrlich, Anupam Mazumdar, Sougato Bose, Carsten Henkel, Ron Folman

**Affiliations:** 1 Department of Physics, Ben-Gurion University of the Negev, 84105 Be’er Sheva, Israel.; 2 Van Swinderen Institute, University of Groningen, 9747 AG Groningen, Netherlands.; 3 Department of Physics and Astronomy, University College London, Gower Street, London WC1E 6BT, UK.; 4 Institute of Physics and Astronomy, University of Potsdam, Potsdam, Germany.

## Abstract

The Stern-Gerlach effect, found a century ago, has become a paradigm of quantum mechanics. Unexpectedly, until recently, there has been little evidence that the original scheme with freely propagating atoms exposed to gradients from macroscopic magnets is a fully coherent quantum process. Several theoretical studies have explained why a Stern-Gerlach interferometer is a formidable challenge. Here, we provide a detailed account of the realization of a full-loop Stern-Gerlach interferometer for single atoms and use the acquired understanding to show how this setup may be used to realize an interferometer for macroscopic objects doped with a single spin. Such a realization would open the door to a new era of fundamental probes, including the realization of previously inaccessible tests at the interface of quantum mechanics and gravity.

## Teaser

A full-loop Stern-Gerlach atom interferometer is realized, opening perspectives to examine the quantum nature of gravity.

## INTRODUCTION

The discovery of the Stern-Gerlach (SG) effect (*1*, *2*) was followed by ideas concerning a full-loop SG interferometer (SGI) consisting of a beam of atoms exposed to field gradients from macroscopic magnets (*3*). However, starting with Heisenberg, Bohm and Wigner (*4*), a coherent SGI was considered impractical because it was thought that the macroscopic device could not be accurate enough to ensure a reversible splitting process, namely, a complete overlap in position and momentum of the two interferometric paths. Bohm (*5*), for example, noted that the magnet would need to have “fantastic” accuracy. Englert, Schwinger, and Scully (*6*–*9*) analyzed the problem in more detail and coined it the Humpty-Dumpty (HD) effect. They too concluded that for substantial coherence to be observed, exceptional accuracy in controlling magnetic fields would be required. While atom interferometers based on light beam splitters enjoy the quantum accuracy of the photon momentum transfer (*10*), the SGI magnets not only have no such quantum discreteness but also suffer from inherent lack of flatness due to Maxwell’s equations. Later work added the effect of dissipation and suggested that only low-temperature magnetic field sources would enable an operational SGI (*11*). Claims have even been made that no coherent splitting is possible at all (*12*).

Here, we provide a detailed account of recent realizations (*13*, *14*) of a full-loop SGI, in which magnetic field gradients act on the atom during its flight through the interferometer as originally envisioned. These realizations build upon recent developments of the spatial fringe SGI (*15*, *16*). In addition, we show that the full-loop SGI can enable a seminal experimental probe: macroscopic-object interferometry.

SG interferometry with mesoscopic objects has been suggested as a compact detector for space-time metric and curvature (*17*), possibly enabling detection of gravitational waves. It has also been suggested as a probe for the quantum nature of gravity (*18*). These SG capabilities may also enable searches for exotic effects like the fifth force, or the hypothesized self-gravitation interaction (*19*–*21*). In the following, we show that such an experiment is, in principle, feasible.

We note that the full-loop SGI configuration (*13*) has already enabled the construction of a unique matter-wave interferometer whose phase scales with the cube of the time that the atom spends in the interferometer (*14*). This realization has been suggested as an experimental test for the Einstein’s equivalence principle when extended to the quantum domain (*22*).

Let us emphasize that a full-loop scheme, with its spin population observable, as described here, has several advantages for macroscopic-object interferometry, over other suggestions that use a spatial interference pattern as their signal. First, a spin population observable requires no high-resolution imaging. For massive objects, this may be crucial. Second, as shown in (*14*), an SGI can achieve a *T*^3^ scaling of the phase accumulation, enabling high sensitivity. Furthermore, the SGI allows us to apply magnetic forces throughout the interferometer, and a significant splitting may be achieved in a few milliseconds or so. Last, and perhaps most importantly, the spin population observable does not require free propagation to develop, in contrast to a spatial interference signal. These reasons provide a crucial advantage when taking into account the high decoherence rate expected for macroscopic objects.

We note that high magnetic stability and accuracy may also make possible technological and metrological applications such as large-area atom interferometry (*23*), sensitive probing of electron transport, e.g., squeezed currents (*24*), as well as nuclear magnetic resonance and compact accelerators (*25*). We note that as the SGI makes no use of light, it may serve as a high-precision surface probe at short distances for which administering light is hard. In addition, our atom chip setup is compatible with cryogenic environments and may hence probe cold surfaces, while laser light may cause unwanted heating. Last, know-how described in this work may assist to advance also other interferometric configurations that use static fields, e.g., electric fields (*26*, *27*).

### The full-loop SGI

In the following, we describe how we obtain a high full-loop SGI contrast of 95% by using the particularly accurate magnetic fields of an atom chip (*28*). Following the footsteps of impressive endeavors (*29*–*39*), and additional recent scientific advancements with spatial SGIs (*15*, *16*), this full-loop SGI (*13*, *14*) is, to the best of our knowledge, the first realization of a complete SGI analogous to that originally envisioned.

**Fig. 1:**
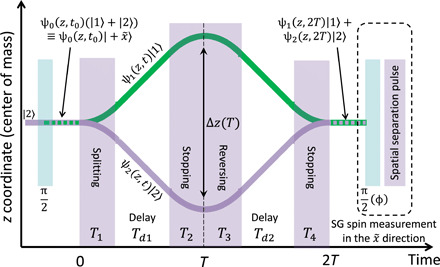
The longitudinal full-loop SGI (*z* position versus time) in the center-of-mass frame. The interferometer operates for a duration of 2*T* (on the order of a few hundred microseconds) and consists of four magnetic gradient pulses (purple columns). The states ∣1〉 ≡ ∣*F* = 2, *m_F_* = 1〉 and ∣2〉 ≡ ∣2,2〉 are defined along the $\tilde{z}$ axis in the Bloch sphere (different from real-space coordinates). The signal is made of spin population fringes. The experiment starts with a spin in the $\tilde{x}$ direction, and the final measurement is again of the spin in the $\tilde{x}$ direction. The latter is performed by mapping the spins from $\tilde{x}$ to $\tilde{z}$ with a π/2 rotation and applying a SG gradient to separate the populations before taking an image. The same configuration may be used for a macroscopic-object interferometer; see the “Testing different aspects of gravity” section.

**Fig. 2:**
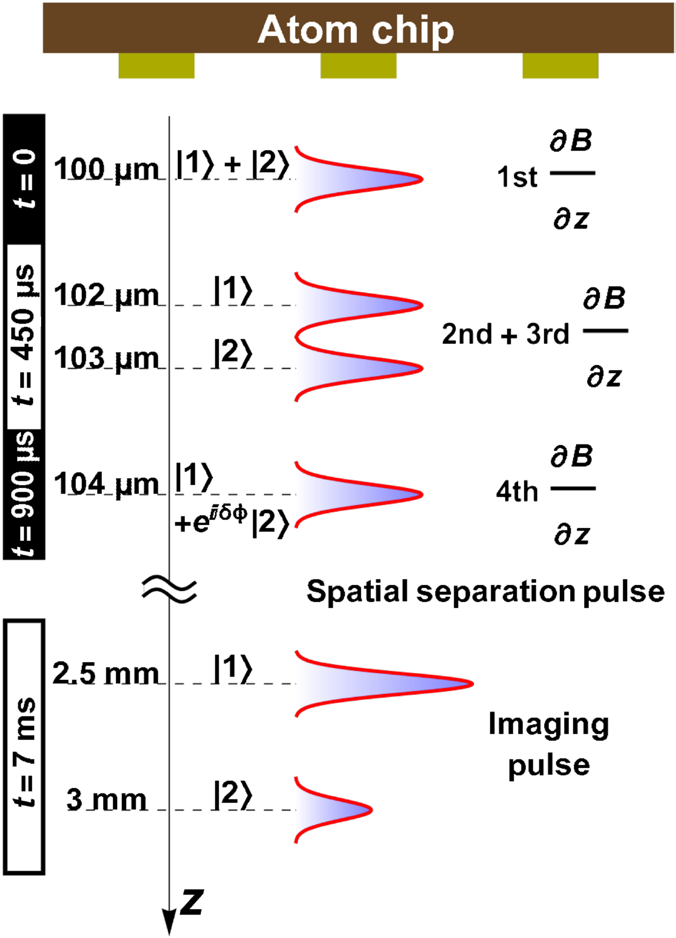
The longitudinal full-loop SGI (*z* position versus time) in the laboratory frame (along the axis of gravity). In addition to the pull of gravity, the wave packets are being accelerated and decelerated by the magnetic gradient pulses. The full-loop sequence is finished after the fourth gradient, after which the spatial separation and imaging pulses are applied to measure the different spin populations of states ∣1〉 and ∣2〉, thus performing a measurement of the spin in the $\tilde{x}$ direction (combined with a leading π/2 pulse; see Fig. 1). A similar configuration may be used for a macroscopic-object interferometer; see the “Testing different aspects of gravity” section. The only difference between the atomic configuration shown here and the macroscopic-object configuration presented in the following is in the final measurement. Measuring the spin state of a single macroscopic object does not use spatial splitting and is simply done by optical readout (e.g., as in done with nanodiamond spins).

A schematic of the full-loop interferometer is presented in Fig. 1 in the center-of-mass frame and in Fig. 2 in the laboratory frame, along the axis of gravity. The full-loop SGI uses four magnetic gradient regions or pulses: splitting, stopping, accelerating back, and stopping, the latter two closing a loop in the space-time diagram. To the best of our knowledge, all previous SG realizations used only the first one or two operations, thereby realizing at most a “half loop.” The entanglement of spin with the spatial degrees of freedom persists throughout the SGI, and the magnetic moment associated with the spin is used to control the external degrees of freedom, using magnetic gradients and the SG effect. The SGI actively recombines the wave packets in both position and momentum and uses the spin state of the recombined wave packet as an interference signal. This is in contrast to the spatial fringe SGI realized in our previous work (*15*, *16*), which consists only of splitting and stopping the wave packets (thus corresponding to a half loop, to which we added spin mixing to allow for spatial fringes to form, in analogy to a double-slit experiment).

The recombination in the full-loop SGI is, in fact, required to be a time-reversal operation of the splitting process, such that the final two magnetic gradients exactly undo the first two. To obtain high coherence (or contrast) in the output of a spatial interferometer, one must apply stable and accurate operations on the atom, such that the final relative distance between the wave packets’ centers, Δ*z*(2*T*), and the final relative momentum between the centers, Δ*p*(2*T*), are minimized, where 2*T* is the interferometer duration. Inaccuracy of the magnetic field gradient throughout the particle’s trajectory gives rise to imperfect overlap, either in position or momentum, and will cause a decay in the resulting interferometric contrast.

The difficulty in maintaining spin coherence due to inaccuracy is illustrated by the following simple argument relating to the momentum splitting [as argued by Heisenberg and others (*4*, *6*, *40*)]. To achieve macroscopic splitting of the wave packets using a differential force *F* acting for a duration *T*_1_, the relative momentum between the split wave packets *FT*_1_ must be much bigger than the initial wave packet width in momentum σ*_p_*, i.e., *FT*_1_ ≫ σ*_p_*. As each part of the wave packet samples a different part of the potential, it acquires a different phase. The linear phase spread over the wave packet is thus given by ∣δϕ∣=∣δ(*ET*_1_/ħ)∣=∣(∂*E*/∂*z*)σ*_z_T*_1_/ħ∣= *FT*_1_σ*_z_*/ħ ≫ σ*_p_*σ*_z_*/ħ, where σ*_z_* is the initial wave packet width in position (σ*_p_* and σ*_z_* are both defined in the *z* direction) and *E* is the atom’s energy due to the magnetic field. By invoking the uncertainty principle, one finds that δϕ may not be made small, further complicating the recombination, where a successful recombination requires maximizing the overlap integral of the two wave packets. In other words, large splitting requires large relative momentum in units of the internal momentum width, which corresponds to a large phase spread over the wave packet (due to the relation between momentum and phase—*e*^*ipz*/ħ^). To achieve high coherence, this phase spread originating from momentum splitting has to be undone. If the size of the two wave packets is the same, then minimizing Δ*p* ensures to some degree that the phase profile of both wave packets is the same, which is sufficient. Note that, in a practical experiment, the phase pattern is actually more complex and harder to match because of the curvature of the magnetic potential.

The precision with which one has to recombine the wave packets is set by the spatial coherence length *l_z_* and momentum coherence width *l_p_*, which we use as a phenomenological model to describe the loss of contrast. These measures of coherence are inversely proportional to the momentum and position uncertainties of the atom, σ*_p_* and σ*_z_*, and may be defined as (*41*)

$$l_{z}=\frac{\hbar }{\sigma_{p}},\;l_{p}=\frac{\hbar }{\sigma_{z}}$$ (1)

where the momentum and position uncertainties satisfy the uncertainty relation σ*_p_*σ*_z_* ≥ ħ/2. If the two paths at the output port of the interferometer (time *t* = 2*T*) are displaced by a distance Δ*z*(2*T*), then the contrast is expected to drop as $C\propto \text{exp}\;[-\frac{1}{2}{\left(\Delta z(2T)/l_{z}\right)}^{2}]$. Equivalently, if the two paths are displaced in momentum by Δ*p*(2*T*), then the contrast reduces as $C\propto \text{exp}\;[-\frac{1}{2}{\left(\Delta p(2T)/l_{p}\right)}^{2}]$.

In the case of a minimal-uncertainty Gaussian wave packet with a negligible expansion rate, the loss of contrast (or coherence) *C* due to inaccuracy of the recombination process is quantified by the HD equation, which is given by (*7*)

$$C=\text{exp}\;[-\frac{1}{8}{\left(\frac{\Delta z(2T)}{\sigma_{z}}\right)}^{2}-\frac{1}{8}{\left(\frac{\Delta p(2T)}{\sigma_{p}}\right)}^{2}]$$ (2)

(Note a factor ^1^/_2_ in our definition of Δ*z* and Δ*p* relative to the original definition.) This equation is the result of calculating the overlap integral between the Gaussian wave functions at the end of the interferometer. To keep the contrast close to unity, a “microscopic” level of accuracy is required at the end of the interferometer, described by the relations ∣Δ*z*(2*T*)∣ ≪ σ*_z_* and ∣Δ*p*(2*T*)∣ ≪ σ*_p_*. Quantitatively, to maintain a contrast of ≃0.99 using *FT*_1_/σ*_p_* = 10^3^, it turns out that one must control the fields with an accuracy of δ*F*/*F* = 10^−5^ (*6*, *7*), a formidable technical challenge. Addressing this challenge is expected to open the door to a wide variety of new experiments for technology and fundamental studies.

### Experiment

Our experiment begins by releasing a Bose-Einstein condensation (BEC) of about 10^4 87^Rb atoms from a magnetic trap below an atom chip (*28*). We initially prepare the BEC in the state ∣*F*, *m_F_*〉 = ∣2,2〉 and then create a superposition of the two spin states ∣*F*, *m_F_*〉 = ∣2,2〉 ≡ ∣2〉 and ∣2,1〉 ≡ ∣1〉 by applying a π/2 radio frequency (RF) pulse. These two states constitute an effective two-level system, as all other states in the *F* = 2 manifold are pushed out of resonance by the nonlinear Zeeman shift generated using an external bias field (see Methods for more details). To avoid dephasing of the spin superposition due to noise in the bias fields, we add π pulses giving rise to an echo sequence (see Methods). The full-loop SGI is then applied by using a series of four magnetic gradient pulses (gradients along the axis of gravity, *z*; see Fig. 1), which are generated by running currents on the atom chip [more details on the setup can be found in (*16*)]. The first pulse, of duration *T*_1_, splits the superposition into two momentum components, which then freely propagate during a delay time *T*_*d*1_. The wave packets are then stopped relative to one another (pulse duration *T*_2_), accelerated back (pulse duration *T*_3_), and, after a second delay time *T*_*d*2_, are stopped again (pulse duration *T*_4_), ideally when overlap in space and momentum is maximal. As the direction of acceleration in the second and third pulses is opposite to that of the first and fourth pulses, we name the *T*_2_ and *T*_3_ pulses the reverse pulses. As the *T*_3_ and *T*_4_ pulses are required to undo the splitting in position and momentum created by the *T*_1_ and *T*_2_ pulses, *T*_3_ and*T*_4_ are named the recombination pulses. We obtain the population signal with the help of a second π/2 pulse followed by a spin population measurement. We measure the visibility by scanning the phase ϕ of the second π/2 pulse (Fig. 1) and observe the contrast of the resulting population fringes.

**Fig. 3:**
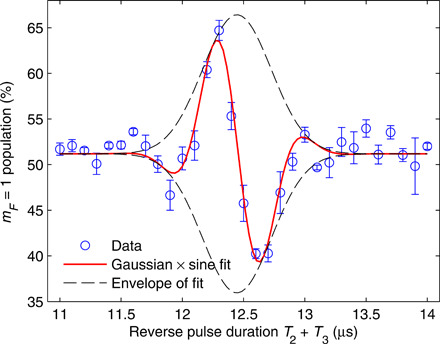
Full-loop optimization procedure: Population output as a function of the reverse pulse duration *T*_2_ + *T*_3_, for *T*_1_ = *T*_4_ = 6 μs, *T*_*d*1_ = 300 μs, and a relative acceleration *a* = 635 m/s^2^. The population oscillates around the optimal point as expected by a simplified model of a Gaussian times a sine. The peak of the Gaussian envelope corresponds to the time at which the wave packets’ overlap integral at the end of the interferometer is maximized for the given parameters. The sine function corresponds to the added phase between the two interferometer arms, per unit time of reversing pulse.

To minimize Δ*z*(2*T*) and Δ*p*(2*T*) (inaccuracies in the final recombination), and thus maximize the visibility of the interference signal, we must optimize the experimental parameters. In Fig. 3, we present an example optimization run, in which we fix the durations of the first and last gradient pulses *T*_1_ and *T*_4_ and also the durations of the delay times *T*_*d*1_ and *T*_*d*2_ (usually *T*_1_ = *T*_4_ and *T*_*d*1_ = *T*_*d*2_ = *T_d_* to begin with). We measure the output population as a function of the duration *T*_2_ + *T*_3_ of the second and third gradient (reverse) pulses, while keeping the total duration *T*_2_ + *T*_3_ + *T*_*d*2_ constant. The data nicely fit a Gaussian envelope times a sine function. The peak of the envelope corresponds to the point in which the overlap integral is maximal. Ideally, for linear magnetic gradients, one would expect the peak overlap to occur when the sequence is symmetric, i.e., *T*_1_ + *T*_4_ = *T*_2_ + *T*_3_ (assuming *T*_*d*1_ = *T*_*d*2_). However the nonlinearity of the magnetic potential created by the chip wires in the *z* direction (*16*), together with the wave packets’ movement with respect to the chip, breaks the symmetric timing diagram. The optimal duration of the gradient pulses depends on the delay times, the initial distance from the chip, and the specific scheme used (see Methods for more details about the optimization procedure). Generally, one needs to optimize two independent parameters—one to minimize the final relative position Δ*z*(2*T*) and one to minimize the final relative momentum Δ*p*(2*T*).

While, as noted in the introduction, accuracy is the main challenge, we also need to address the issue of stability, whereby temporal fluctuations may give rise to dephasing, decoherence, and drifts. Even in the absence of decoherence, noise may cause the interference phase to jitter from one experimental shot to the next (e.g., due to a fluctuating bias field), thus dephasing the averaged phase or preventing recombination in the case of a different noise (e.g., fluctuating gradients). In this work, we achieve high stability by using an atom chip (*28*) with several advantages, including strong magnetic gradients so that the experimental duration is very short and, consequently, the interaction with external noise is brief and very low inductance so that the gradients can be switched in microseconds. In addition, the structure and position of the magnet are very precise as it is made of a near-perfect wire. Also, care was taken to reduce a wide range of hindering effects. For example, a novel method is used to reduce the effect of current fluctuations on the chip by using a three-wire configuration that produces a quadrupole and exposes the wave packets to a weaker magnetic field from the chip, while maintaining strong gradients. Using these advantages, we have been able to show low-phase noise (*16*) (SD as low as 0.1 rad of the spatial fringe SGI), demonstrating the stability of the apparatus.

## RESULTS

**Fig. 4:**
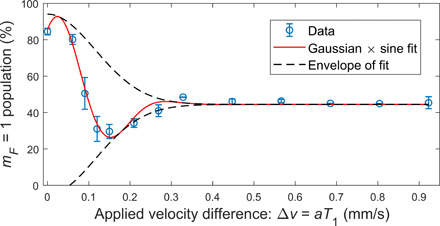
Single kick effect on the contrast: Population as a function of the applied velocity difference Δ*v* between the wave packets for a single pulse of duration *T*_1_ (where we set *T*_2_ = *T*_3_ = *T*_4_ = 0). As the splitting is increased, the population decays to 50%—corresponding to zero contrast. In this measurement, we take care that the spatial separation is as small as possible (Δ*z* ≤ 50 nm) such that the decay due to spatial splitting is negligible. The velocity difference is calculated according to Δ*v* = *aT*_1_, where *a* = 59.5 m/s^2^ is the applied relative acceleration. The data are fitted to a Gaussian times a sine function, and the fit returns a momentum coherence width of *l_p_*/*m* = 0.12 ± 0.03 mm/s, where *m* is the atom’s mass. To the best of our knowledge, this is the first direct measurement of the momentum coherence width of a BEC [see previous results using neutrons (*76*) and atomic beams (*77*)].

**Fig. 5:**
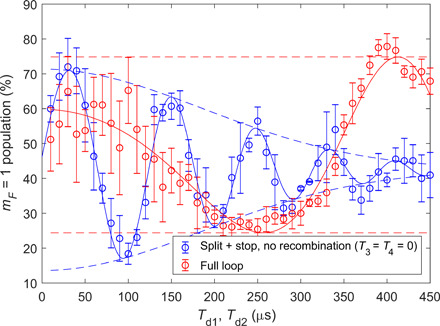
Full-loop recombination: Population in the *m_F_ =* 1 state as a function of delay times *T_d_* for two different sequences, using *T*_1_ *=* 5.4 μs and a relative acceleration *a =* 367 m/s^2^. Blue data are an optimized splitting (*T*_1_) and stopping (*T*_2_) sequence without recombination (i.e., *T*_3_, *T*_4_ = 0). The optimization procedure ensures that the visibility loss due to momentum splitting is minimized for every value of *T*_*d*1_, meaning that we see visibility loss mainly due to spatial splitting (as it becomes large relative to the coherence length). The red data use the same parameters of *T*_1_, *T*_*d*1_, and *T*_2_ but add the recombining pulses. Here, we use *T*_*d*1_ = *T*_*d*2_ = *T_d_*, and they are scanned together. Solid lines are fits to the data (see main text for details); dashed lines represent the envelope of the fit, demonstrating the visibility as a function of the delay time. Note that the red data seem to start with a lower contrast. This effect of flatness and “increasing contrast” is expected by the theory (*14*) and originates from a nonlinear phase term. One can clearly see that for long delay times, the spatial splitting reduces the visibility of the interference. The recombination then revives the visibility, demonstrating successful recombination in both position and momentum. An independent measurement of the contrast at *T*_*d*1_ = *T*_*d*2_ = 450 μs gives the value *C* = 0.48 ± 0.05, thus validating that the contrast is high (see methods for details).

We now validate that we are able to successfully recombine in position and momentum, as both can cause loss of visibility. First, in Fig. 4, we present the loss of coherence due to the first magnetic pulse alone (i.e., by setting *T*_2_, *T*_3_, *T*_4_ = 0), giving rise to orthogonality in momentum. The contrast drops to $1/\sqrt{e}$ at *l_p_*/*m* = 0.12 mm/s momentum splitting. Next, we apply a much stronger momentum splitting of Δ*p*/*m* = 2.6 mm/s and show in Fig. 5 (blue data) that we are able to undo this orthogonality by a second gradient pulse, which stops the relative motion of the wave packets. However, as we extend the delay time *T*_*d*1_, the wave packets start to split in space and we observe a decay in visibility that cannot be restored using *T*_2_ alone, as the distance between the wave packets becomes larger than their coherence length *l_z_*. We validate that this loss of visibility is mainly due to spatial splitting, by optimizing *T*_2_ for each value of *T*_*d*1_, such that maximal visibility is achieved (see Methods for details).

Using this method, we are able to also accurately determine *l_z_*. To describe the expected loss of contrast with increasing spatial splitting, we fit the blue data with a Gaussian times a sine function of the form $P_{1}=A\text{exp}\;(-\frac{1}{2}T_{d}^{2}/\tau^{2})\text{sin}\;[\delta \phi (T_{d})+\phi_{0}]+c$, where δϕ(*T_d_*) is the accumulated phase difference, which contains terms proportional to *T_d_* and $T_{d}^{2}$ (*14*), ϕ_0_ is a constant phase shift, *A* is the amplitude, *c* is a constant, and τ, the decay constant, is essentially the coherence time (as *T_d_* is much larger than the pulse time). Using the value of τ, we calculate the spatial coherence length *l_z_* and obtain *l_z_* = 0.38 ± 0.08 μm (see Methods). Note that the blue data decay to less than the expected 50% value, probably because of imperfect RF π/2 pulses, affecting the state preparation and population measurement.

We are now ready to recombine the two wave packets. We add the recombining pulses *T*_3_ and *T*_4_ to the previous sequence and generate the red data in Fig. 5, where the value of *T*_4_ is also optimized in a similar manner for maximal visibility. One can clearly see the Gaussian decay of the visibility due to spatial splitting in the first (blue) data and the revival of the visibility due to the successful recombination of the spatially split wave packets (red data). At *T*_*d*1_ = *T*_*d*2_ = 350 μs, for example, the blue data decay to 16% of their original amplitude, while the red data show no decay at this time scale. The red data are fitted with a similar function as that used for the blue data, but without the decaying Gaussian term (as the decay is not visible in this range), namely, *P*_1_ = *A* sin [δϕ(*T_d_*) + ϕ_0_] + *c*.

In summary, we have clearly shown the successful recombination in momentum and position, thus realizing a complete SGI. We have also measured *l_p_* and *l_z_*, and the observed visibility is proof that the accuracy of our recombination in momentum and position is better than these coherence scales (see Methods for more discussion on the results).

### Limits on accuracy

**Fig. 6:**
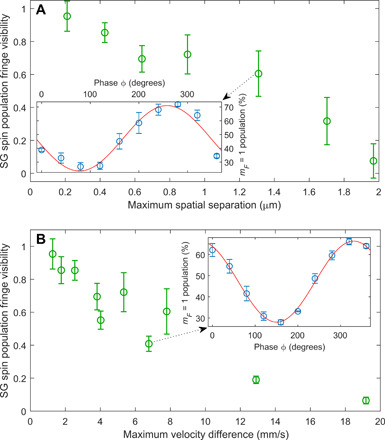
Analysis of accuracy. (**A**) Spin population visibility versus maximal separation Δ*z*(*T*). Experimental parameters are *T*_1_ = 2 − 30 μs, *T*_*d*1_ ≃ *T*_*d*2_ = 50 − 160 μs. (**B**) Visibility versus maximal momentum splitting Δ*p*(*T*_1_). Experimental parameters are *T*_1_ = 2 − 6 μs, *T*_*d*1_ ≃ *T*_*d*2_ = 164 − 367 μs. In both (A) and (B), the relative acceleration is varied in the range *a* = 635 − 2641 m/s^2^. As expected, coherence decays as we increase spatial and momentum splitting. A detailed quantitative analysis could not explain the observed slope (see Discussion). In both panels, the visibility is normalized to the Ramsey visibility without splitting (i.e., no magnetic gradients), typically ~90%. Insets show the raw data for two of the data points, namely, the population fringe generated using a phase scan of the second π/2 pulse and a fit to a sine wave used to extract the contrast (red line; see Methods). Data points in (A) and (B) are of different datasets.

To test the limits of our accuracy, in Fig. 6, we plot the visibility as a function of the maximum splitting in momentum Δ*p*(*T*_1_) and in position Δ*z*(*T*). To try and maximize the splitting, we use several different configurations: We invert the sign of the relative acceleration by reversing the sign of the currents in the chip wires, while, in other sequences, we keep the same currents while inverting the spins with the help of π pulses (see Methods). We also use a variety of magnetic gradient magnitudes by varying the current on the chip and scan both the splitting gradient pulse duration *T*_1_ and the delay time between the pulses *T_d_*. Each point in Fig. 6 was taken using different parameters and was optimized independently. For weak splitting, we observe high visibility (∼95%), while, for a momentum splitting equivalent to 1 ħ*k* (optical photon recoil on the rubidium D2 line, ħ*k*/*m* = 5.8 mm/s, where *m* is the atom’s mass), the visibility is still high (∼75%), indicating that the magnetic field accuracy allowed reversing the splitting to a high degree. The quoted values are normalized to that of a pure Ramsey sequence, i.e., a sequence without any magnetic gradients, to cancel technical effects (see Methods). The visibility as a function of maximum splitting is qualitatively the same for space and momentum splitting and shows a decrease in visibility as splitting is increased.

As we know the potential created by the chip, the wave packets’ positions and widths can be calculated in every step of the experiment. The HD equation (Eq. 2) should thus, in principle, allow for a quantitative calculation of the contrast using the experimental parameters. However, the equation makes two simplifying assumptions: first, that the wave packets do not expand during the propagation through the interferometer, which does not hold in our case. Second, it assumes that there is no nonlinear phase imprinted on the wave packets (i.e., magnetic gradients are linear). In our case, phase nonlinearity originates both from the curvature of the magnetic gradient generated by the chip and from conversion of the mean-field potential energy of the BEC released from trap to kinetic energy [causing a quadratically varying phase to evolve (*42*)]. To account for these effects and try to quantitatively explain the loss of contrast in the full-loop SGI, we have developed a generalized wave packet model for studying coherence of matter-wave interferometers (*43*). While we have simulated our experimental conditions with care, and while these simulations accurately describe our previous interferometry results [e.g., (*14*–*16*, *44*, *45*)], the coherence drop observed in the full-loop experiment is not well described by the generalized wave packet model (*43*), and neither by the HD model. Using the experimental parameters to calculate the visibility, both models predict values that are significantly higher than those observed in the experiment. We therefore leave the quantitative comparison to future work.

To give limits on our accuracy in recombining the wave packets, we use the experimentally measured coherence scales as a figure of merit and examine the ratio between the achieved maximum splittings and the coherence scales. We are able to recombine wave packets with a maximum spatial splitting of Δ*z*(*T*)/*l_z_* = 5.1 before visibility goes to zero and a maximum momentum splitting of Δ*p*(*T*_1_)/*l_p_* = 158. These results indicate that we are more successful in recombining in momentum than in space. The reason for this difference is yet unclear.

## DISCUSSION

We now discuss the possibility of testing different aspects of gravity using the full-loop SGI. Macroscopic quantum states have been of great interest for a long time (*46*). While some proposals exist with the goal of probing gravity with atoms (*20*, *21*, *47*, *48*), here, we aim at using a superposition of a macroscopic object. The idea of using the SGI with a macroscopic object as a probe for gravity has been described in detail in several theoretical works (*17*, *18*, *22*, *49*–*51*). These works detail a wide range of experiments from detection of gravitational waves to the testing of the quantum nature of gravity. In the following, we discuss the feasibility of such an experiment using the full-loop SGI, as described above for an atom, and show that such an experiment using a macroscopic body is also feasible. As before, our interference observable is oscillations of spin population, rather than spatial fringes (density modulations). This observable, as demonstrated in the atomic SGI described above, has the advantages that there is no requirement for long evolution times to allow the spatial fringes to develop, there is no need for high-resolution imaging to resolve the spatial fringes, and the phase accumulation may be fast [e.g., *T*^3^ (*14*)]. Let us note that there are other proposals to realize a spatial superposition of macroscopic objects (*52*, *53*), but they do not rely on the SG effect, and they use spatial rather than spin population fringes.

The masses that one would like to consider at first are in the 10^6^ to 10^10^ atom range. Let us first emphasize that, even before any probing of gravity, a successful SGI will already achieve at least three orders of magnitude more atoms than the state of the art in macroscopic-object interferometry (*54*), thus contributing novel insight to the foundations of quantum mechanics. Another contribution to the latter would be the ability to test continuous spontaneous localization models [e.g., (*55*, *56*) and references therein].

When considering gravity, the first contribution of such a massive-object SGI would simply be to measure small *g* (the local gravitational acceleration). Such an experiment would first of all enable to verify the created superposition of the macroscopic object (*49*, *57*, *58*). It should be emphasized that the interferometer is expected to have a unique *T*^3^ signature (*14*), which would ensure that a spatial superposition was achieved.

A second contribution of such an SGI in the field of gravity would be in testing modifications to gravity at short ranges (also known as the fifth force), as one of the SGI paths may be brought close to a massive object, thus allowing to probe gravity at short distances (*59*, *60*). This is a regime in which light-based interferometers would be difficult to use because of diffraction and light scattering near the surface. Once SGI technology allows for large masses, a third contribution will be the testing of hypotheses concerning gravity self-interaction (*19*–*21*), and when large-area SGI with large masses is available, a fourth contribution would be to detect gravitational waves (*17*). Last, it is claimed that placing two such SGIs in parallel next to each other will allow probing the quantum nature of gravity (*18*, *51*, *61*). An important point here is that, already at 10^9^ atoms, the gravitational interaction becomes stronger than the spin-spin interaction at a distance of 100 μm. We note that (*61*) also talks of 10^−16^ kg, which is less than 10^10^ atoms. Let us emphasize that although high accelerations may be obtained with multiple spins, in the following, we discuss only the case of a macroscopic object with a single spin, as the observable of such a quantum-gravity experiment is entanglement between two spins, and averaging over many spins may wash out the signal. Furthermore, a multispin SGI would give rise to multiple trajectories.

We focus solely on the required SG parameters, which include the necessary accelerations and accuracy in relation to the required splitting and coherence length, respectively. Other issues such as material science (e.g., clean surfaces against patch potentials, spin contaminations, or spin coherence time near the surface), or the very good vacuum that will be required, will not be dealt with here. We will also not deal with the issue of the Casimir-Polder or diamagnetic interactions that may arise. These issues have been dealt with extensively in previous works (*18*, *61*). While we believe that all discussed applications noted here, including the two parallel interferometers for the quantum-gravity experiment, may be done in the longitudinal configuration presented in the experimental part of this paper, transverse [i.e., two-dimensional (2D)] interferometers may also be realized with the same techniques discussed here. Both the 1D and 2D interferometers involve similar operations and present the same challenges, and in the following, we will not discuss the differences between them.

We consider, for example, a nanodiamond composed of 10^6^ carbon atoms (corresponding to a sphere radius of 11 nm) with a single nitrogen-vacancy (NV) spin embedded in it. Coherence time of 1 ms has been demonstrated at room temperature (*62*) and 0.6 s while cooling to 77 K (*63*). These times are for bulk. It may very well be that reasonable coherence times will only be achieved at a distance greater than, say, 20 nm from the surface, in which case the lowest feasible nanodiamond size has 10^7^ atoms. A diameter of about 40 to 50 nm also fits well with lithographic resolutions, which enable to create such nanodiamonds in a controlled manner through the etching of pillars from bulk. These structures have already exhibited a room-temperature coherence time of 200 μs (*64*). It is clear that, in any case, significant material engineering will be required. The total interferometer time of the proposed experiment will have to be shorter than these times. Using well-known NV techniques [see, for example, our own work (*65*, *66*)], we do not see any fundamental spin-related obstacles.

The experimental procedure is the same as for the atomic full-loop SGI demonstrated above, with the required adjustments for manipulating the nanodiamond. The experiment begins by trapping and cooling the center-of-mass motion of the nanodiamond (*67*, *68*). We note that, to the best of our knowledge, ground-state cooling of nanodiamonds is yet to be achieved (*69*). We then release the nanodiamond from the trap and prepare it in a spin superposition of the ±1 spin projections using a π/2 microwave pulse (a two-photon transition). Placing the nanodiamond 1 μm away from a 1 × 1 μm^2^ wire on an atom chip, carrying 1 A of current [with a current density of 10^8^ A/cm^2^, achievable with carbon nanotube–embedded Cu wires (*70*)], we get a magnetic gradient of 8.7 × 10^4^ T/m. For 10^6^ carbon atoms, the acceleration for a single spin (1 Bohr magneton) is *a* = μ*_B_∂_z_B*/*m* = 81 m/s^2^. We then apply a microwave π pulse to inverse the relative acceleration and apply the stopping and reversing pulses *T*_2_ and *T*_3_. After another π pulse, we apply *T*_4_ and stop the relative motion (see Fig. 1 for description of pulses). For total interferometer times of *T* = (0.1,1,10,100) ms, we get maximum splittings of Δ*z* = *a*(*T*/4)^2^ = 5 × (10^−8^,10^−6^,10^−4^,10^−2^) m. Maintaining a constant acceleration for the long durations requires placing current-carrying wires along the wave packets’ trajectories such that they are always in a region of strong gradient [e.g., as previously suggested in (*17*)] or realizing the experiment in a very strong quadrupole field created by coils (e.g., superconducting). We note that, if the nanodiamond is very close to the wire, then one of the spin states will have to be 0 so that it does not crash into the wire. The spin state is then read out by standard NV optical techniques. Let us briefly recall that the phase is predicted to be independent of the initial motional condition (*49*).

The crucial parts of the experiment are the initial cooling and the recombination. The overlap integral, which determines the interferometer visibility (the HD effect), depends on both, and it will only give a significant nonzero value if the coherence length (a function of temperature) is better than the recombination imprecision (Eq. 2). One should therefore aspire to achieve ground-state cooling.

The coherence length is given by the harmonic oscillator length $\sqrt{\hbar /m\omega }$, which, for the considered object, may be assumed to be about 0.1 nm [assuming ground-state cooling with ω/2π = 80 kHz, as in (*67*)]. This value can be increased by adiabatically lowering the trap frequencies after cooling. We do not take into account some works that claim that techniques exist with which the coherence length may be increased even further (*53*, *71*), as these are done for spatial interference fringes, where the “local” coherence length is what matters (*71*). In contrast, what matters for the full-loop SGI is the overlap integral, for which the coherence length only depends on the initial momentum width, namely, on the ground state energy when cooling to the ground state.

The recombination accuracy represents the main technical challenge, as it must be better than the achieved coherence length. In the experimental results presented above for an atomic SGI, high visibility is achieved for a 700-nm coherence length, which allows us to assume that we have obtained a recombination accuracy on the order of 100 nm (when the maximal splitting was an order of magnitude larger). Improving this recombination accuracy by three orders of magnitude (while maintaining the same ratio to the maximal splitting) is well within reach by using better current sources with less current and time jitters. (We note that at present, we use current sources with instabilities far above shot noise.)

Let us briefly also touch upon the topic of decoherence due to external and internal degrees of freedom, namely, which-path information due to some scattering event between the environment and the nanodiamond. The information may be encoded in the environment [i.e., the scattered particle, equivalent to the interaction with some noise with a correlation length smaller than the spatial splitting (*72*)] or in the internal degrees of freedom. For example, as explained in (*73*), any object with excited internal degrees of freedom may emit radiation that would localize it. Other sources of decoherence may originate from any differential interaction that the nanodiamond suffers between the two paths.

As a simple example of lack of decoherence due to the internal degrees of freedom, we can consider the atom interferometer demonstrated in this paper. The atom has many internal degrees of freedom such as those of the electrons or the nucleus. Decoherence can occur because of the emergence of which-path information, or, in other words, orthogonality. However, if the inner degrees of freedom do not develop some orthogonality along the two paths, then they are irrelevant for the interferometer, as no which-path information is encoded in them. What can be relevant is some differential interaction between those internal degrees of freedom and the environment that can create such orthogonality, where, by differential, we mean that the interaction with the environment affecting the internal degrees of freedom, in a coordinate system relative to the center of mass (c.o.m), is different for the two paths of the interferometer. In the case of atom interferometry, it seems that external perturbations are negligible with respect to the atom’s bare Hamiltonian.

In contrast, with the nanodiamond, the differential interaction between the internal degrees of freedom and the environment may not be negligible. For example, a background gas collision can introduce rotation or phonons in the nanodiamond, in only one of the paths. The collision can even be with a cosmic muon. Furthermore, an external magnetic/electric/electromagnetic field that creates rotation or phonons, or some spin flip of a contamination spin, just in one of the paths (due to a small correlation length), would give rise to orthogonality. A more subtle process of decoherence would be the excitation of phonons, solely due to the magnetic force acting on the NV centers. Here, the sudden (nonadiabatic) force acting on the spin would create a phonon in the lattice (this may be estimated through a Debye-Waller factor). As the acceleration in the two paths is different, the excited phonons would be different, giving rise to orthogonality. Even if the acceleration is symmetric but opposite in sign, the opposite *k*-vector of the identical phonons would create orthogonality. To maintain adiabaticity, one may have to resort to engineering a gradual increase of the magnetic gradient within the pulse duration.

Calculating the cross section/probability for these events and the amount of orthogonality that they create is a daunting task. Some of these calculations have already been done in (*18*, *53*) and references therein. In any case, we do not see a clear correlation between these cross sections and the internal temperatures, and so a priori, there is no clear need for the cooling of the internal degrees of freedom or the environment, as long as the wavelength of the blackbody radiation is larger than the splitting induced in the SGI. If, however, for some reason, cooling of the internal degrees of freedom is required, then the experiment may be done in a dilution fridge cryostat, with which the atom chip technology is compatible. Additional methods to avoid decoherence, such as dynamic decoupling, have been suggested as well (*74*). In any case, the fact that the SGI can achieve large spatial splitting in a very short time, as presented above, is a crucial advantage when addressing the issue of decoherence.

Last, let us emphasize that the SGI also has two clear advantages over laser-pulse interferometers in the context of such an experiment: (i) Laser-pulse matter-wave beam splitters require an appropriate internal transition to coherently scatter photons; this demand severely restricts the applicability of these splitters to macroscopic objects. The SGI merely requires a magnetic moment, which enables to readily achieve the magnetomechanical coupling. (ii) No heating is generated on the macroscopic object because no light is scattered. The scattering of light may also severely reduce the amount of light interacting with the two-level system embedded in the object. Lack of light scattering also suppresses the decoherence rate.

To conclude, we have analyzed in detail recent realizations of a full-loop SGI, consisting of freely propagating atoms exposed to magnetic gradients, as originally envisioned decades ago. We have unambiguously shown recombination in both momentum and position. We have shown that SG splitting may be realized in a highly coherent manner with macroscopic magnets without requiring cryogenic temperatures or magnetic shielding. Furthermore, we have analyzed the limits of our system’s accuracy.

We briefly compare our experiments to state-of-the-art SG interferometry (*29*–*39*). While these longitudinal beam experiments did show spin population interference fringes, the experiments presented here are very different. As explained in (*34*) and (*37*), the full-loop configuration was never realized, as only splitting and stopping operations were applied (i.e., no active recombination); namely, wave packets exit the interferometer with the same separation as the maximal separation achieved within.

Last, achieving this high level of control over magnetic gradients may facilitate fundamental research as well as technological applications. Specifically, we show that, in principle, full-loop SG interferometry with a macroscopic body is feasible. It may be used to test the foundations of quantum theory, as well as to probe exotic forces such as the fifth force. It may be developed as a gravitational sensor, serve to test exotic gravitational models such as self-interaction, and, lastly, may probe the quantum nature of gravity.

## METHODS

### Detailed experimental scheme

**Fig. 7:**
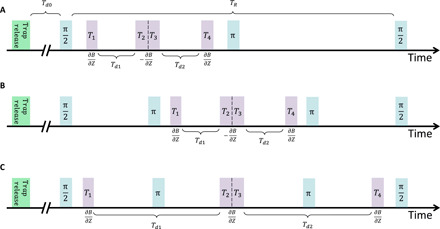
Timing diagram of the full-loop experimental sequences. (**A** and **B**) The current inversion scheme, where the sign of the gradient ∂B/*∂z* is switched during the sequence. The difference between (A) and (B) is the additional π pulse before the gradients, used to increase the coherence time. (**C**) The spin inversion scheme, in which the sign of the gradient *∂B*/*∂z* is kept constant, and the relative force between the spin states is inverted by using the π pulses in between the gradients, inverting the spin states.

In the following, we describe our experimental sequence. Initial steps are similar to the spatial fringe SGI, and experimental setup is the same as used in (*16*). We begin by preparing a BEC of about 10^4 87^Rb atoms in the state ∣*F*, *m_F_* > =∣2,2> in a magnetic trap located around 91 − 96 μm ±1 μm below the atom chip surface (different experiments use different initial positions). The harmonic frequencies of the trap are ω*_x_*/2π = 38 Hz and ω*_y_*/2π ≈ ω*_z_*/2π = 127 Hz. The trap is created by a copper structure located behind the chip with the help of additional homogeneous bias magnetic fields in the *x*, *y*, and *z* directions. The BEC is then released from the trap and falls a few micrometers under gravity for a duration *T*_*d*0_ = 0.9 to 1.4 ms (see Fig. 7 for a timing diagram). During this time, the magnetic fields used to generate the trap are turned off completely. Only a homogeneous magnetic bias field of 36.7 G in the *y* direction is kept on to create an effective two-level system via the nonlinear Zeeman effect such that the energy splitting between our two levels ∣2,2 > ≡ ∣2> and∣2,1 > ≡ ∣1> is *E*_21_ ≈ *h*×25 MHz, and where the undesired transition is off-resonance by *E*_21_ − *E*_10_ ≈ *h*×180 kHz. As the BEC expands, interaction becomes negligible, and the experiment may be described by single-atom physics.

Next, we apply an RF π/2 pulse (10-μs duration) to create an equal superposition of the two spin states, ∣1> and ∣2>, and a magnetic gradient pulse (splitting pulse) of duration *T*_1_ = 4 to 40 μs, which creates a different magnetic potential for the different spin states *m_F_*, thus splitting the atomic wave packet into two wave packets with different momenta. The chip wire current is driven using simple 12-V batteries connected in series and is modulated using a home-made current shutter. The SG acceleration is in the range of 59.5 to 2641 m/s^2^ (depending on chip current). The acceleration is measured by splitting the wave packets using a single pulse and measuring the relative distance as a function of the time of flight (*15*).

After a delay time *T*_*d*1_, we apply a stopping pulse of duration *T*_2_ that cancels the relative momentum of the two wave packets, and immediately after a gradient pulse for accelerating the atoms back toward each other (*T*_3_). After an additional delay time *T*_*d*2_, we apply a final stopping pulse (*T*_4_) so that the two wave packets overlap in momentum and position.

A second RF π/2 pulse is applied only at the time of measurement (meaning that the two wave packets have a different spin throughout the propagation), which completes the interferometric sequence. Without the magnetic gradient pulses and their effect on the spatial wave function, the two π/2 pulses correspond to a Ramsey sequence. Our signal is an interference pattern formed by measuring the spin population (e.g., starting with $S_{\tilde{x}}=+1$ and measuring $S_{\tilde{z}}$).

The visibility, or contrast, which represents spin coherence in our experiment, is measured by changing the phase between the two interferometer arms and observing the resulting population oscillation between the two states. In some measurements, we scan the magnetic gradient pulse duration, thus adding a relative phase between the arms (*14*) (as in Figs. 3 and 5).

In other measurements in which the gradient pulse durations are fixed, we create a population interference pattern by shifting the phase ϕ of the last RF π/2 relative to the first RF π/2 pulse. This creates population oscillations between the states (as a function of ϕ), generating the oscillations shown in the insets of Fig. 6. Contrast is then evaluated by fitting the population fringes to a function of the form *P*(ϕ) = 0.5*C* sin (ϕ + ϕ_0_) + const, where *C* is the contrast, ϕ is the applied phase shift, and ϕ_0_ is a constant phase term. As noted, the resulting contrast shown in Fig. 6 is normalized to that of a pure Ramsey sequence, i.e., a sequence without any magnetic gradients. This cancels technical effects that reduces the contrast, such as nonzero RF detuning (causing imperfect π/2 pulses), spin decoherence due to external magnetic noise, etc.

As noted before, we add one or two RF π pulses in between the two π/2 pulses, giving rise to an echo sequence that suppresses the dephasing taking place because of magnetic noise and inhomogeneous magnetic fields in our chamber. This allows us to increase the spin coherence time from ∼400 μs up to ∼4 ms.

Relative populations in each output port are measured by applying a homogeneous magnetic gradient that separates the spin states and counting the number of atoms in each output state using standard absorption imaging. The homogeneous gradient is created by running a current in the copper structure behind the chip for a few milliseconds.

Last, it is worth noting that while all gradient pulses come from the same chip wires, the magnetic pulses may be considered as an analogy of the original thought experiment in which there were different spatial regions with different permanent magnets. This is so as in each pulse the current and duration may be different and have individual jitter, and in addition, the atom position and consequently the gradient are different.

### Full-loop configurations

Here, we describe in more detail the “current inversion” and “spin inversion” sequences shown in a timing diagram in Fig. 7, which are used to generate the data in Fig. 6. In the first sequence, after applying a π/2 pulse and the splitting gradient *T*_1_ in one direction (downward toward gravity), we reverse the sign of the acceleration by reversing the sign of the currents in the chip wires for the stopping and reversing gradients *T*_2_ and *T*_3_, working in the opposite direction (upward toward the chip; this is done using two independent current shutters connected to the chip in opposite directions). The opposite gradient causes the relative movement between the wave packets to stop during *T*_2_ and to change sign during *T*_3_. Last, the wave packets are brought to a relative stop and spatial overlap by the second stopping pulse *T*_4_ given in the same direction as the first gradient. The sequence is finished by applying a π pulse (to increase coherence time, giving rise to an echo sequence) and a π/2 pulse, for mixing the different spin states and enabling spin populations interference (the π/2 − π − π/2 sequence is symmetric in time). The four consecutive gradient pulses used in the current inversion sequence are applied either after the first π/2 pulse (i.e., only a single π pulse is used as described above) or in between two π pulses to further increase the coherence time (the π/2 − π − π − π/2 sequence is again time symmetric).

In the second sequence, we keep the same current direction in all gradient pulses, while reversing the spins with the help of two π pulses. These pulses are applied, first just before the stopping gradient pulse *T*_2_ and second just after the reversing gradient pulse *T*_3_. Each π pulse causes the spin states to flip sign, thus changing the direction of the applied momentum kicks in the center-of-mass frame (in laboratory frame, all gradient pulses push the atoms downward toward gravity). Both the current inversion and spin inversion sequences are used to generate the data points for Fig. 6.

### Minimizing the visibility loss due to momentum splitting

In Fig. 5, we validate that the loss of visibility is due to spatial splitting, by minimizing the visibility loss due to momentum splitting for each value of *T*_*d*1_, using the following optimization procedure. We scan for the optimal value of *T*_2_, i.e., the value that obtains the maximal visibility, for several delay times (*T*_*d*1_ = 100,200,300,400 μs), in a similar way to what is shown in Fig. 3. We then determine the optimal *T*_2_ for any given value of *T*_*d*1_ using a polynomial interpolation. The blue data in Fig. 5 are taken by using the optimal values of *T*_2_ as a function of *T*_*d*1_. This ensures that the visibility loss due to momentum splitting is minimized for every value of *T*_*d*1_, meaning that we see visibility loss mostly due to spatial splitting.

**Fig. 8:**
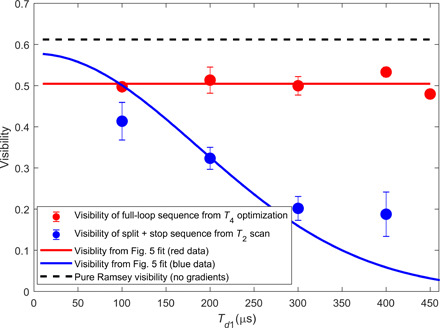
Visibility comparison: Split + stop sequence and full-loop recombination sequence. Data points show the visibility obtained from the optimization sequence for each sequence (see methods for details). Red and blue lines show the visibility values obtained from the fit to the data in Fig. 5 of the corresponding sequences. The high values of the red data demonstrate the effective recombination and validate the values shown in Fig. 5. Dashed line shows the visibility of the pure Ramsey sequence (i.e., without any gradients) as a reference. The limited visibility is due to inaccurate RF π/2 pulses (detuning), caused mainly by the varying distance between the atoms and the antenna.

To validate the contrast shown in Fig. 5, in Fig. 8, we compare the visibility values obtained from the fit to the data in Fig. 5 to the values extracted from the optimization procedure. The observed agreement confirms that the recombination revives the visibility, thus validating successful recombination in both position and momentum.

### Calculation of *l_z_*

In the fit of the blue data of Fig. 5, *T_d_* = τ represents the time at which the contrast drops to $1/\sqrt{e}$ of its full value. Accordingly, we estimate the spatial coherence length *l_z_* from the spatial splitting at *T_d_* = τ. This is the sum of three contributions: (i) the splitting under constant acceleration *a* for a duration *T*_1_, given by $\frac{1}{2}{\mathrm{aT}}_{1}^{2}$; (ii) the splitting at constant velocity *aT*_1_ for a duration *T_d_*, given by *aT*_1_*T_d_*; and (iii) the splitting under constant deceleration −*a*, from initial velocity *aT*_1_ until zero relative velocity, for duration *T*_2_ = *T*_1_, given by ${\mathrm{aT}}_{1}T_{1}-\frac{1}{2}{\mathrm{aT}}_{1}^{2}=\frac{1}{2}{\mathrm{aT}}_{1}^{2}$. Summing all contributions and setting *T_d_* = τ, we obtain $l_{z}={\mathrm{aT}}_{1}^{2}+{\mathrm{aT}}_{1}\tau$. Using the experimental parameters *a* = 367 ± 61 m/s^2^, *T*_1_ = 5.4 μs, and the fit result of τ = 186.8 ± 22.5 μs, we obtain *l_z_* = 0.38 ± 0.08 μm.

### Coherence scales

Here, we compare the experimental results of the coherence scales to those obtained from Eq. 1. Assuming that the BEC is a minimal-uncertainty wave packet, at trap release time, both its coherence scales are set by a single number—the size of the BEC wave packet. We therefore begin by calculating this number and validating it experimentally. After some time of flight, however, the BEC momentum width increases because of conversion of the mean-field potential energy to kinetic energy (*42*), which we account for using the theory of (*43*).

The in-trap Thomas-Fermi condensate half-length *w*_0_ in the *z* (gravity) direction is given by (*75*) $w_{0}=\sqrt{2\mu /m}/\omega_{z}$, where the Thomas-Fermi expression for the chemical potential μ for an harmonically confined condensate is given by (*75*) $\mu^{5/2}=15\hbar^{2}m^{1/2}N{\bar{\omega }}^{3}a/2^{5/2}$, and where $\bar{\omega }={\left(\omega_{x}\omega_{y}\omega_{z}\right)}^{1/3}$ is the geometric mean of the trap frequencies, *N* is the number of atoms, and *a* = 5.18 nm is the ^87^Rb s-wave scattering length. For our experimental parameters of *N* = 10,000 atoms, ω_*x*, *y*_/2π = 40 Hz and ω*_z_*/2π = 126 Hz, we obtain *w*_0_ = 2.88 μm.

We also experimentally validate this value: As imaging the wave packet at short time-of-flight *T*_*d*0_ (see Fig. 7) does not give reliable results because of refraction effects around a BEC with high optical density, we measure the Thomas-Fermi wave packet size as a function of *T*_*d*0_ and use the known trap frequency ω*_z_* to calculate *w*_0_ according to $w(T_{d0})=w_{0}\sqrt{1+\omega_{z}^{2}T_{d0}^{2}}$ (*75*). This method gives the value *w*_0_ = 3.04 ± 0.3 μm in reasonable agreement with the theoretically calculated value.

As the HD theory and the generalized wave-packet model (*43*) both assume a Gaussian wave packet, we express the Thomas-Fermi size in terms of Gaussian width by fitting it to a Gaussian profile, which gives σ*_z_* ≃ 0.41*w*_0_ = 1.2 μm for the in-trap size. Also taking into account the experimental time of flight of *T*_*d*0_ = 1 ms (i.e., the time difference between trap release and the start of the SGI sequence), the wave packet size at the start of the SGI sequence is $\sigma_{z}(t=0)=1.2\;\mu m\times \sqrt{1+\omega_{z}^{2}T_{d0}^{2}}=1.5\;\mu m$.

The measured values of *l_p_* and *l_z_* can now be compared to theory (Eq. 1) using the obtained BEC wave packet size. A momentum coherence width of *l_p_*/*m* = 0.12 mm/s corresponds to a wave packet size of σ*_z_* = 6.1 μm. This means that the momentum coherence width is about 4.1 times narrower than expected for a pure condensate. The discrepancy could originate from the contribution of the thermal fraction of the atoms, although we evaluate the BEC fraction to be larger than 0.7, so we cannot fully explain this effect yet.

A coherence length of *l_z_* = 0.38 μm corresponds to a momentum uncertainty of σ*_p_*/*m* = 1.9 mm/s, while the expected momentum uncertainty of a pure BEC after 1-ms expansion is σ*_p_*/*m* ≈ 0.6 mm/s (including increased momentum width due to conversion of the mean-field potential energy to kinetic energy). This means that the coherence length is shorter than expected for a pure condensate, a discrepancy that is only partially explained by the thermal fraction of atoms.

## References

[1] W. Gerlach, O. Stern, Der experimentelle Nachweis der Richtungsquantelung im Magnetfeld. Z. Phys. 9, 349–352 (1922).

[2] M. O. Scully, W. E. Lamb Jr., A. Barut, On the theory of the Stern-Gerlach apparatus. Found. Phys. 17, 575–583 (1987).

[3] E. P. Wigner, The problem of measurement. Am. J. Phys. 31, 6–15 (1963).

[4] H.J. Briegel, B.-G. Englert, M.O. Scully, H. Walther, in *Atom Interferometry*, P. R. Berman, Ed. (Academic Press, 1997), p. 240.

[5] D. Bohm, *Quantum Theory* (Prentice-Hall, 1951).

[6] B.-G. Englert, J. Schwinger, M. O. Scully, Is spin coherence like Humpty-Dumpty? I. Simplified treatment. Found. Phys. 18, 1045–1056 (1988).

[7] J. Schwinger, M. O. Scully, B.-G. Englert, Is spin coherence like Humpty-Dumpty? II. General theory. Z. Phys. D 10, 135–144 (1988). 10.1103/physreva.40.1775 9902333

[8] M. O. Scully, B.-G. Englert, J. Schwinger, Spin coherence and Humpty-Dumpty. III. The effects of observation. Phys. Rev. A 40, 1775–1784 (1989). 10.1103/physreva.40.1775 9902333

[9] B.-G. Englert, Time reversal symmetry and Humpty-Dumpty. Z. Naturforsch. A 52, 13–14 (1997).

[10] A. D. Cronin, J. Schmiedmayer, D. E. Pritchard, Optics and interferometry with atoms and molecules. Rev. Mod. Phys. 81, 1051–1129 (2009).

[11] T. R. de Oliveira, A. O. Caldeira, Dissipative Stern-Gerlach recombination experiment. Phys. Rev. A 73, 042502 (2006).

[12] M. Devereux, Reduction of the atomic wavefunction in the Stern–Gerlach magnetic field. Can. J. Phys. 93, 1382–1390 (2015).

[13] Y. Margalit, Z. Zhou, O. Dobkowski, Y. Japha, D. Rohrlich, S. Moukouri, R. Folman, Realization of a complete Stern-Gerlach interferometer. arXiv:1801.02708 [quant-ph] (8 January 2018). 10.1126/sciadv.abg2879 PMC8163084 34049876

[14] O. Amit, Y. Margalit, O. Dobkowski, Z. Zhou, Y. Japha, M. Zimmermann, M. A. Efremov, F. A. Narducci, E. M. Rasel, W. P. Schleich, R. Folman, *T*^3^ Stern–Gerlach matter-wave interferometer. Phys. Rev. Lett. 123, 083601 (2019). 31491196 10.1103/PhysRevLett.123.083601

[15] S. Machluf, Y. Japha, R. Folman, Coherent Stern–Gerlach momentum splitting on an atom chip. Nat. Comm. 4, 2424 (2013). 10.1038/ncomms3424 24013518

[16] Y. Margalit, Z. Zhou, S. Machluf, Y. Japha, S. Moukouri, R. Folman, Analysis of a high-stability Stern–Gerlach spatial fringe interferometer. New J. Phys. 21, 073040 (2019).

[17] R. J. Marshman, A. Mazumdar, G. W. Morley, P. F. Barker, S. Hoekstra, S. Bose, Mesoscopic interference for metric and curvature & gravitational wave detection. New J. Phys. 22, 083012 (2020).

[18] S. Bose, A. Mazumdar, G. W. Morley, H. Ulbricht, M. Toros, M. Paternostro, A. A. Geraci, P. F. Barker, M. S. Kim, G. Milburn, Spin entanglement witness for quantum gravity. Phys. Rev. Lett. 119, 240401 (2017). 29286711 10.1103/PhysRevLett.119.240401

[19] M. Hatifi, T. Durt, Revealing self-gravity in a Stern-Gerlach Humpty-Dumpty experiment. arXiv:2006.07420 [quant-ph] (12 June 2020).

[20] R. Howl, R. Penrose, I. Fuentes, Exploring the unification of quantum theory and general relativity with a Bose-Einstein condensate. New J. Phys. 21, 043047 (2019).

[21] I. Fuentes, R. Penrose, Quantum state reduction via gravity, and possible tests using Bose-Einstein condensates, in *Collapse of the Wave Function: Models, Ontology, Origin, and Implications*, S. Gao, Ed. (Cambridge Univ. Press, 2018), pp. 187–206.

[22] C. Marletto, V. Vedral, On the testability of the equivalence principle as a gauge principle detecting the gravitational *t*^3^ phase. Front. Phys. 8, 176 (2020).

[23] P. Hamilton, M. Jaffe, J. M. Brown, L. Maisenbacher, B. Estey, H. Müller, Atom interferometry in an optical cavity. Phys. Rev. Lett. 114, 100405 (2015). 25815912 10.1103/PhysRevLett.114.100405

[24] D. V. Strekalov, N. Yu, K. Mansour, *Sub-Shot Noise Power Source for Microelectronics* (NASA Tech Briefs, 2011); www.techbriefs.com/component/content/article/11341.

[25] E. Danieli, J. Perlo, B. Blümich, F. Casanova, Highly stable and finely tuned magnetic fields generated by permanent magnet assemblies. Phys. Rev. Lett. 110, 180801 (2013). 23683185 10.1103/PhysRevLett.110.180801

[26] J. E. Palmer, S. D. Hogan, Electric rydberg-atom interferometry. Phys. Rev. Lett. 122, 250404 (2019). 31347868 10.1103/PhysRevLett.122.250404

[27] D. Comparat, Limitations for field-enhanced atom interferometry. Phys. Rev. A 101, 023606 (2020).

[28] M. Keil, O. Amit, S. Zhou, D. Groswasser, Y. Japha, R. Folman, Fifteen years of cold matter on the atom chip: Promise, realizations, and prospects. J. Mod. Opt. 63, 1840–1885 (2016). 27499585 10.1080/09500340.2016.1178820 PMC4960518

[29] J. Robert, C. Miniatura, S. Le Boiteux, J. Reinhardt, V. Bocvarski, J. Baudon, Atomic interferometry with metastable hydrogen atoms. Europhys. Lett. 16, 29–34 (1991).

[30] C. Miniatura, F. Perales, G. Vassilev, J. Reinhardt, J. Robert, J. Baudon, A longitudinal Stern–Gerlach interferometer: The “beaded” atom. J. Phys. II 1, 425–436 (1991).

[31] C. Miniatura, J. Robert, S. Le Boiteux, J. Reinhardt, J. Baudon, A longitudinal Stern-Gerlach atomic interferometer. Appl. Phys. B 54, 347–350 (1992).

[32] J. Robert, C. Miniatura, O. Gorceix, S. Le Boiteux, V. Lorent, J. Reinhardt, J. Baudon, Atomic quantum phase studies with a longitudinal Stern-Gerlach interferometer. J. Phys. II 11, 601–614 (1992).

[33] C. Miniatura, J. Robert, O. Gorceix, V. Lorent, S. Le Boiteux, J. Reinhardt, J. Baudon, Atomic interferences and the topological phase. Phys. Rev. Lett. 69, 261–264 (1992). 10046628 10.1103/PhysRevLett.69.261

[34] S. Nic Chormaic, V. Wiedemann, C. Miniatura, J. Robert, S. Le Boiteux, V. Lorent, O. Gorceix, S. Feron, J. Reinhardt, J. Baudon, Longitudinal Stern-Gerlach atomic interferometry using velocity selected atomic beams. J. Phys. B Atom. Mol. Opt. Phys. 26, 1271–1279 (1993).

[35] J. Baudon, R. Mathevet, J. Robert, Atomic interferometry. J. Phys. B Atom. Mol. Opt. Phys. 32, R173–R195 (1999).

[36] M. Boustimi, V. Bocvarski, V. de Lesegno, K. Brodsky, F. Perales, J. Baudon, J. Robert, Atomic interference patterns in the transverse plane. Phys. Rev. A 61, 033602 (2000).

[37] E. Maréchal, R. Long, T. Miossec, J.-L. Bossennec, R. Barbé, J.-C. Keller, O. Gorceix, Atomic spatial coherence monitoring and engineering with magnetic fields. Phys. Rev. A 62, 53603 (2000).

[38] B. Viaris de Lesegno, J. C. Karam, M. Boustimi, F. Perales, C. Mainos, J. Reinhardt, J. Baudon, V. Bocvarski, D. Grancharova, F. Pereira Dos Santos, T. Durt, H. Haberland, J. Robert, Stern Gerlach interferometry with metastable argon atoms: An immaterial mask modulating the profile of a supersonic beam. J. Eur. Phys. J. D 23, 25–34 (2003).

[39] K. Rubin, M. Eminyan, F. Perales, R. Mathevet, K. Brodsky, B. Viaris de Lesegno, J. Reinhardt, M. Boustimi, J. Baudon, J.-C. Karam, J. Robert, Atom interferometer using two Stern-Gerlach magnets. Laser Phys. Lett. 1, 184–193 (2004).

[40] W. Heisenberg, *Die physikalischen Prinzipien der Quantentheorie* (Hirzl, 1930).

[41] J. F. Schaff, T. Langen, J. Schmiedmayer, Interferometry with atoms. La Riv. del Nuovo Cim. 37, 509–589 (2014).

[42] E. W. Hagley, L. Deng, M. Kozuma, M. Trippenbach, Y. B. Band, M. Edwards, M. Doery, P. S. Julienne, K. Helmerson, S. L. Rolston, W. D. Phillips, Measurement of the coherence of a Bose-Einstein condensate. Phys. Rev. Lett. 83, 3122 (1999).

[43] Y. Japha, Generalized wave-packet model for studying coherence of matter-wave interferometers. arXiv:1902.07759 [cond-mat.quant-gas].

[44] Y. Margalit, Z. Zhou, S. Machluf, D. Rohrlich, Y. Japha, R. Folman, A self-interfering clock as a “which path” witness. Science 349, 1205–1208 (2015). 26249229 10.1126/science.aac6498

[45] S. Zhou, D. Groswasser, M. Keil, Y. Japha, R. Folman, Robust spatial coherence 5 μm from a room-temperature atom chip. Phys. Rev. A 93, 063615 (2016).

[46] F. Fröwis, P. Sekatski, W. Dür, N. Gisin, N. Sangouard, Macroscopic quantum states: Measures, fragility, and implementations. Rev. Mod. Phys. 90, 025004 (2018).

[47] R. Howl, V. Vedral, D. Naik, M. Christodoulou, C. Rovelli, A. Iyer, Non-Gaussianity as a Signature of a Quantum Theory of Gravity. PRX Quantum 2, 010325 (2021).

[48] J. Junca, A. Bertoldi, D. O. Sabulsky, G. Lefèvre, X. Zou, J.-B. Decitre, R. Geiger, A. Landragin, S. Gaffet, P. Bouyer, B. Canuel, Characterizing Earth gravity field fluctuations with the MIGA antenna for future gravitational wave detectors. Phys. Rev. D 99, 104026 (2019).

[49] C. Wan, M. Scala, G. W. Morley, A. A. T. M. Rahman, H. Ulbricht, J. Bateman, P. F. Barker, S. Bose, M. S. Kim, Free nano-object ramsey interferometry for large quantum superpositions. Phys. Rev. Lett. 117, 143003 (2016). 27740804 10.1103/PhysRevLett.117.143003

[50] R. J. Marshman, A. Mazumdar, S. Bose, Locality and entanglement in table-top testing of the quantum nature of linearized gravity. Phys. Rev. A 101, 052110 (2020).

[51] C. Marletto, V. Vedral, Gravitationally induced entanglement between two massive particles is sufficient evidence of quantum effects in gravity. Phys. Rev. Lett. 119, 240402 (2017). 29286752 10.1103/PhysRevLett.119.240402

[52] H. Pino, J. Prat-Camps, K. Sinha, B. Venkatesh, O. Romero-Isart, On-chip quantum interference of a superconducting microsphere. Quantum Sci. Technol. 3, 025001 (2018).

[53] O. Romero-Isart, Coherent inflation for large quantum superpositions of levitated microspheres. New J. Phys. 19, 123029 (2017).

[54] Y. Y. Fein, P. Geyer, P. Zwick, F. Kiałka, S. Pedalino, M. Mayor, S. Gerlich, M. Arndt, Quantum superposition of molecules beyond 25 kDa. Nat. Phys. 15, 1242–1245 (2019).

[55] O. Romero-Isart, Quantum superposition of massive objects and collapse models. Phys. Rev. A 84, 052121 (2011).

[56] A. Bassi, K. Lochan, S. Satin, T. P. Singh, H. Ulbricht, Models of wave-function collapse, underlying theories, and experimental tests. Rev. Mod. Phys. 85, 471–527 (2013).

[57] M. Scala, M. S. Kim, G. W. Morley, P. F. Barker, S. Bose, Matter-wave interferometry of a levitated thermal nano-oscillator induced and probed by a spin. Phys. Rev. Lett. 111, 180403 (2013). 24237492 10.1103/PhysRevLett.111.180403

[58] M. Torós, S. Bose, P. Barker, Atom-nanoparticle Schrödinger cats. arXiv:2005.12006 [quant-ph] (25 May 2020).

[59] A. A. Geraci, S. B. Papp, J. Kitching, Short-range force detection using optically cooled levitated microspheres. Phys. Rev. Lett. 105, 101101 (2010). 20867507 10.1103/PhysRevLett.105.101101

[60] D. O. Sabulsky, I. Dutta, E. A. Hinds, B. Elder, C. Burrage, E. J. Copeland, Experiment to detect dark energy forces using atom interferometry. Phys. Rev. Lett. 123, 061102 (2019). 31491160 10.1103/PhysRevLett.123.061102

[61] T. W. van de Kamp, R. J. Marshman, S. Bose, A. Mazumdar, Quantum gravity witness via entanglement of masses: Casimir creening. Phys. Rev. A 102, 062807 (2020).

[62] E. D. Herbschleb, H. Kato, Y. Maruyama, T. Danjo, T. Makino, S. Yamasaki, I. Ohki, K. Hayashi, H. Morishita, M. Fujiwara, N. Mizuochi, Ultra-long coherence times amongst room-temperature solid-state spins. Nat. Commun. 10, 3766 (2019). 31462631 10.1038/s41467-019-11776-8 PMC6713727

[63] N. Bar-Gill, L. Pham, A. Jarmola, D. Budker, R. L. Walsworth, Solid-state electronic spin coherence time approaching one second. Nat. Commun. 4, 1743 (2013). 23612284 10.1038/ncomms2771

[64] M. E. Trusheim, L. Li, A. Laraoui, E. H. Chen, H. Bakhru, T. Schröder, O. Gaathon, C. A. Meriles, D. Englund, Scalable fabrication of high purity diamond nanocrystals with long-spin-coherence nitrogen vacancy centers. Nano Lett. 14, 32–36 (2014). 24199716 10.1021/nl402799u

[65] Y. Rosenzweig, Y. Schlussel, R. Folman, Probing the origins of inhomogeneous broadening in nitrogen-vacancy centers with Doppler-free-type spectroscopy. Phys. Rev. B 98, 014112 (2018).

[66] Y. Schlussel, T. Lenz, D. Rohner, Y. Bar-Haim, L. Bougas, D. Groswasser, M. Kieschnick, E. Rozenberg, L. Thiel, A. Waxman, J. Meijer, P. Maletinsky, D. Budker, R. Folman, Wide-field imaging of Superconductor vortices with electron spins in diamond. Phys. Rev. Appl. 10, 034032 (2018).

[67] U. Delić, M. Reisenbauer, K. Dare, D. Grass, V. Vuletić, N. Kiesel, M. Aspelmeyer, Cooling of a levitated nanoparticle to the motional quantum ground state. Science 367, 892–895 (2020). 32001522 10.1126/science.aba3993

[68] F. Tebbenjohanns, M. Frimmer, V. Jain, D. Windey, L. Novotny, Motional sideband asymmetry of a nanoparticle optically levitated in free space. Phys. Rev. Lett. 124, 013603 (2020). 31976693 10.1103/PhysRevLett.124.013603

[69] J.-F. Hsu, P. Ji, C. W. Lewandowski, B. D’Urso, Cooling the motion of diamond nanocrystals in a magneto-gravitational trap in high vacuum. Sci. Rep. 6, 30125 (2016). 27444654 10.1038/srep30125 PMC4957077

[70] C. Subramaniam, T. Yamada, K. Kobashi, A. Sekiguchi, D. N. Futaba, M. Yumura, K. Hata, One hundred fold increase in current carrying capacity in a carbon nanotube–copper composite. Nat. Comm. 4, 2202 (2013). 10.1038/ncomms3202 PMC3759037 23877359

[71] D. E. Miller, J. R. Anglin, J. R. Abo-Shaeer, K. Xu, J. K. Chin, W. Ketterle, High-contrast interference in a thermal cloud of atoms. Phys. Rev. A 71, 043615 (2005).

[72] A. Stern, Y. Aharonov, Y. Imry, Phase uncertainty and loss of interference: A general picture. Phys. Rev. A 41, 3436–3448 (1990). 9903511 10.1103/physreva.41.3436

[73] K. Hornberger, J. E. Sipe, M. Arndt, Theory of decoherence in a matter wave Talbot-Lau interferometer. Phys. Rev. A 70, 053608 (2004).

[74] J. S. Pedernales, G. W. Morley, M. B. Plenio, Motional dynamical decoupling for interferometry with macroscopic particles. Phys. Rev. Lett. 125, 023602 (2020). 32701327 10.1103/PhysRevLett.125.023602

[75] W. Ketterle, D. S. Durfee, D. M. Stamper-Kurn, Making, probing and understanding Bose-Einstein condensates, in *Proceedings of the International School of Physics “Enrico Fermi”*, M. Inguscio, S. Stringari, C. E. Wieman, Eds. (IOS Press 1999), vol. 140, pp. 67–176.

[76] R. Golub, S. K. Lamoreaux, Elucidation of the neutron coherence length and a matter-wave sideband interferometer. Phys. Lett. A 162, 122–128 (1992).

[77] E. T. Smith, A.-A. Dhirani, D. A. Kokorowski, R. A. Rubenstein, T. D. Roberts, H. Yao, D. E. Pritchard, Velocity rephased longitudinal momentum coherences with differentially detuned separated oscillatory fields. Phys. Rev. Lett. 81, 10 (1996).

